# Development of a Novel, Multi-Parametric, MRI-Based Radiomic Nomogram for Differentiating Between Clinically Significant and Insignificant Prostate Cancer

**DOI:** 10.3389/fonc.2020.00888

**Published:** 2020-06-30

**Authors:** Yongsheng Zhang, Wen Chen, Xianjie Yue, Jianliang Shen, Chen Gao, Peipei Pang, Feng Cui, Maosheng Xu

**Affiliations:** ^1^The First Clinical Medical College of Zhejiang Chinese Medical University, Hangzhou, China; ^2^Department of Radiology, The Guangxing Hospital Affiliated to Zhejiang Chinese Medical University, Hangzhou, China; ^3^Department of Radiology, The First Affiliated Hospital of Zhejiang Chinese Medical University, Hangzhou, China; ^4^GE Healthcare Life Sciences, Hangzhou, China

**Keywords:** prostate cancer, magnetic resonance imaging, radiomic, nomogram, prediction

## Abstract

**Objectives:** To develop and validate a predictive model for discriminating clinically significant prostate cancer (csPCa) from clinically insignificant prostate cancer (ciPCa).

**Methods:** This retrospective study was performed with 159 consecutively enrolled pathologically confirmed PCa patients from two medical centers. The dataset was allocated to a training group (*n* = 54) and an internal validation group (*n* = 22) from one center along with an external independent validation group (*n* = 83) from another center. A total of 1,188 radiomic features were extracted from T2WI, diffusion-weighted imaging (DWI), and apparent diffusion coefficient (ADC) images derived from DWI for each patient. Multivariable logistic regression analysis was performed to develop the model, incorporating the radiomic signature, ADC value, and independent clinical risk factors. This was presented using a radiomic nomogram. The receiver operating characteristic (ROC) curve was utilized to assess the predictive efficacy of the radiomic nomogram in both the training and validation groups. The decision curve analysis was used to evaluate which model achieved the most net benefit.

**Results:** The radiomic signature, which was made up of 10 selected features, was significantly associated with csPCa (*P* < 0.001 for both training and internal validation groups). The area under the curve (AUC) values of discriminating csPCa for the radiomics signature were 0.95 (training group), 0.86 (internal validation group), and 0.81 (external validation group). Multivariate logistic analysis identified the radiomic signature and ADC value as independent parameters of predicting csPCa. Then, the combination nomogram incorporating the radiomic signature and ADC value demonstrated a favorable classification capability with the AUC of 0.95 (training group), 0.93 (internal validation group), and 0.84 (external validation group). Appreciable clinical utility of this model was illustrated using the decision curve analysis for the nomogram.

**Conclusions:** The nomogram, incorporating radiomic signature and ADC value, provided an individualized, potential approach for discriminating csPCa from ciPCa.

## Introduction

Prostate cancer (PCa) is the second most frequently diagnosed cancer in men worldwide (1). The serum prostate-specific antigen (PSA) and digital rectal examination are the most widely used in the PCa screenings in clinical practice (2). If a patient presents with an elevated PSA, transrectal ultrasound (TRUS)-guided biopsy is the conventional diagnostic approach. However, about over 30% of men undergo side effects with TRUS-guided biopsy, including pain, bleeding infection, and hematuria, and ~1% need to be hospitalized for observation (3). Furthermore, some patients experience unnecessary biopsies as clinically insignificant PCa (ciPCa), defined as a Gleason score (GS) <3+4 or a maximum cancer core length of <6 mm, may be detected (4). The clinically significant PCa (csPCa) is defined as a GS ≥ 3+4 in at least one biopsy core pathology (4–6). The principal treatment of ciPCa is active surveillance rather than radical prostatectomy, which is routine treatment for localized csPCa. In addition, the detection of ciPCa by transrectal ultrasound-guided biopsy may cause overtreatment in a few patients.

Multi-parametric MRI (mp-MRI) containing anatomical sequences (T1- and T2-weighted imaging; T1WI and T2WI) and functional sequences [diffusion-weighted imaging (DWI) and dynamic contrast-enhanced (DCE)] has been regarded as an advanced imaging pattern in the identification of PCa (7, 8). Mp-MRI plays an important role in decreasing the overdiagnosis and overtreatment for ciPCa, arranging target biopsy, tumor stage, or treatment for csPCa patients. However, its diagnostic performance and evaluation capacity varies based on each individual radiologist. The overall inter-reader consistency of multiple reports ranges from poor (0.5) to moderate (0.71), mainly depending on the experience and learning level of radiologists (9, 10).

Radiomic methods are regarded as a noninvasive, efficient, and reliable method for adopting advanced image-processing techniques to extract a variety of quantitative features from imaging data (11). Radiomics has been mainly used in oncology, for instance, lung cancer, brain astrocytoma, and breast carcinoma, wherein radiomics is utilized to identify tumor stage, curative effect, prognosis assessment, and genetic analysis (12–14). Radiomics has also been extended to PCa, mainly focusing on PCa diagnosis and differentiation (15–18). Min et al. investigated an mp-MRI-based radiomic signature for predicting patients with csPCa (18). The results showed that the radiomic signature had a potential to discriminate csPCa from ciPCa, wherein the area under the curve (AUC) was 0.823 in the validation cohort. However, the diagnostic efficacy of an mp-MRI-based radiomic nomogram in the identification of csPCa has not been completely determined. The use of nomograms has been widely accepted as a reliable method for determining quantitative risk factors for clinical events (19). In this study, we hypothesized that a radiomic nomogram incorporating an mp-MRI-based radiomic signature and independent clinical risk factors can non-invasively discriminate csPCa from ciPCa in patients with suspected PCa. Therefore, we sought to develop and validate a radiomic nomogram that would incorporate a radiomic signature and clinical risk factors for the pre-biopsy prediction of csPCa.

## Materials and Methods

### Patient Cohort

**Figure 1:**
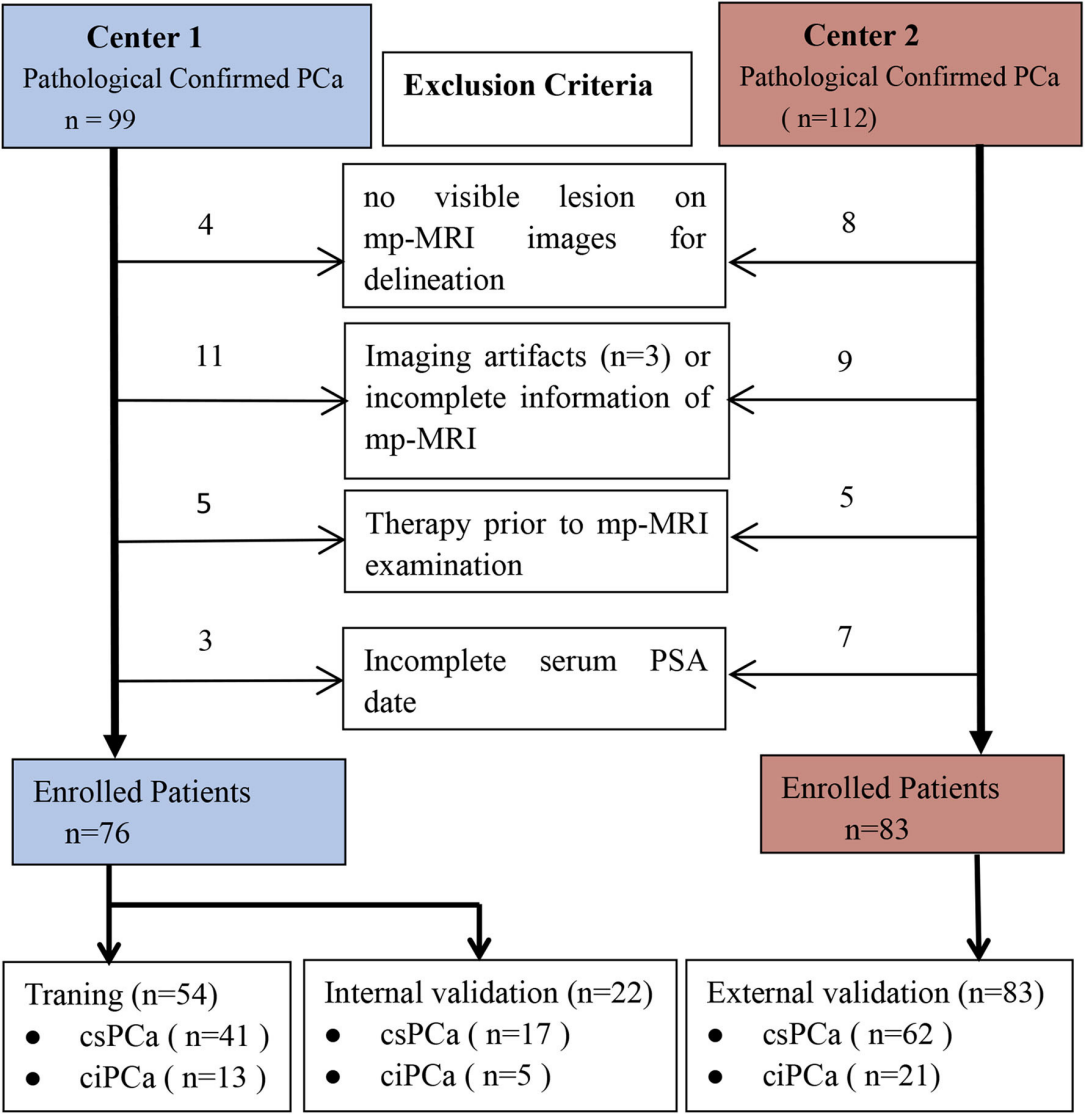
Diagram for inclusion of patients into the study. csPCa, clinically significant prostate cancer; ciPCa, clinically insignificant prostate cancer; mp-MRI, multi-parametric MRI; PSA, prostate-specific antigen.

This retrospective study was approved by the Institutional Ethical Committee of the Guangxing Hospital Affiliated to Zhejiang Chinese Medical University and the First Affiliated Hospital of Zhejiang Chinese Medical University, which waived the requirement for written informed consent. The study consecutively enrolled 159 patients with biopsy pathology-proven PCa who received mp-MRI examination from January 2016 to February 2020. All patients were scanned on the same model scanner and did not receive TRUS-guided biopsy prior to MRI examination. Exclusion criteria were (1) prior therapy history for PCa patients including antihormonal therapy, radiation, cryotherapy, or prostatectomy; (2) incomplete information or severe imaging artifacts of the MRI images; (3) lesion diameter <5 mm on mp-MRI images; and (4) lack of serum PSA level (Figure 1). The enrolled patients were randomly assigned to a training group (*n* = 54) and an internal validation group (*n* = 22) from the Guangxing Hospital Affiliated to Zhejiang Chinese Medical University along with an external independent validation group (*n* = 83) from the First Affiliated Hospital of Zhejiang Chinese Medical University another center.

Baseline clinical features were derived from medical records, including age and PSA level with the cutoff value of 10 ng/ml. The interval time between MRI and PSA testing was less than 1 month.

### MRI Examination

All recruited patients were scanned using the same model 3.0 T MRI (Discovery 750W 3.0T, GE Healthcare, Milwaukee, USA) with a 32-channel pelvic coil. The protocol included transverse T1WI; transverse, sagittal, and coronal T2WI; transverse DWI; apparent diffusion coefficient (ADC) imaging derived from DWI; and dynamic contrast-enhanced. DWI was applied with a *b* value of 0 s/mm^2^, 1000 s/mm^2^. The details of the imaging sequence parameters of two medical centers are summarized in Supplementary Table 1.

### Lesion Segmentation on MR Images

Only T2WI, DWI, and ADC images were incorporated in this study because of the availability and emphasis in Prostate Imaging and Reporting and Data System version 2(PI-RADS v2) (7). The software package ITK-SNAP (version 3.4.0; www.itksnap.org) was used for manual segmentation of PCa lesion. The region of interest (ROI) was delineated along the boundaries of the lesion layer by layer in reference to the biopsy's pathological results. Given the importance of heterogeneity analysis, ROI was designed to contain regions of calcification, necrosis, bleeding, and cystic tissue, not including structures such as the urethra, seminal vesicle, and other normal anatomical structures. For differing pathological GSs, the highest biopsy GS regions were uniquely selected for delineation. If all lesions demonstrated the same GS on multi-focal PCa, the ROIs were depicted at each level manually until all lesions were incorporated.

A radiologist (W.C. with 3 years of experience of abdominal MRI) who was blind to the GS of each PCa lesion measured ADC value. The ROIs were placed to comprise as much of the inner aspect of the lesion as possible without encompassing surrounding normal structure on the ADC map. There was between one and three ROIs of each patient with a mean area of 40 mm^2^ (range, 10–80 mm^2^). Another abdomen radiologist (F.C. with 21 years of experience of abdominal MRI) who was blind to the PCa lesion evaluated the MR-T stage for each patient in reference to NCCN guidelines (20).

### Intra- and Inter-observer Agreement

The intra- and inter-observer agreements for feature extraction were assessed by the intra-class correlation coefficient (ICC). Initially, integrated imaging data of 20 patients were randomly selected from the study group. All ROIs on T2WI, DWI, and ADC images were rigorously outlined with the same criteria by two experienced radiologists independently. Intra-observer ICC was analyzed by comparing two extractions of reader 1 (Y.Z. with 10 years' experience of abdominal MRI). Inter-observer ICC was evaluated by comparing the extraction of a second reader (F.C. with 21 years' experience of abdominal MRI) and the extraction of reader 1. An ICC that was >0.8 was regarded as a good agreement and the remaining image segmentation was implemented by reader 1 (21).

### Radiomic Feature Extraction and Model Building

AK software (Artificial Intelligence Kit V3.0.0.R, GE Healthcare) was performed to extract a total of 396 radiomic features per ROI of each MRI scan, including the histogram, second-order statistic, Gray-Level Co-occurrence Matrix (GLCM), Run length matrix (RLM), and form factor parameters (15). The histogram, also called first-order statistic, represents the distribution of values of each voxel without concern for spatial relationships. The second-order statistic was routinely named as the texture features, which described the statistical relationships between voxels with similar (or dissimilar) contrast values. The overall number of the radiomic features in this study was 1,188. Before feature selection, the values of individual feature for the whole patients was normalized with *Z*-scores ((x–μ)/σ), wherein x is the value of the feature, μ represents the mean values of this feature for all patients in the set, and σ describes the corresponding standard deviation so as to get rid of the unit limits of each feature prior to being performed for a machine learning model for classification (22).

**Figure 2:**
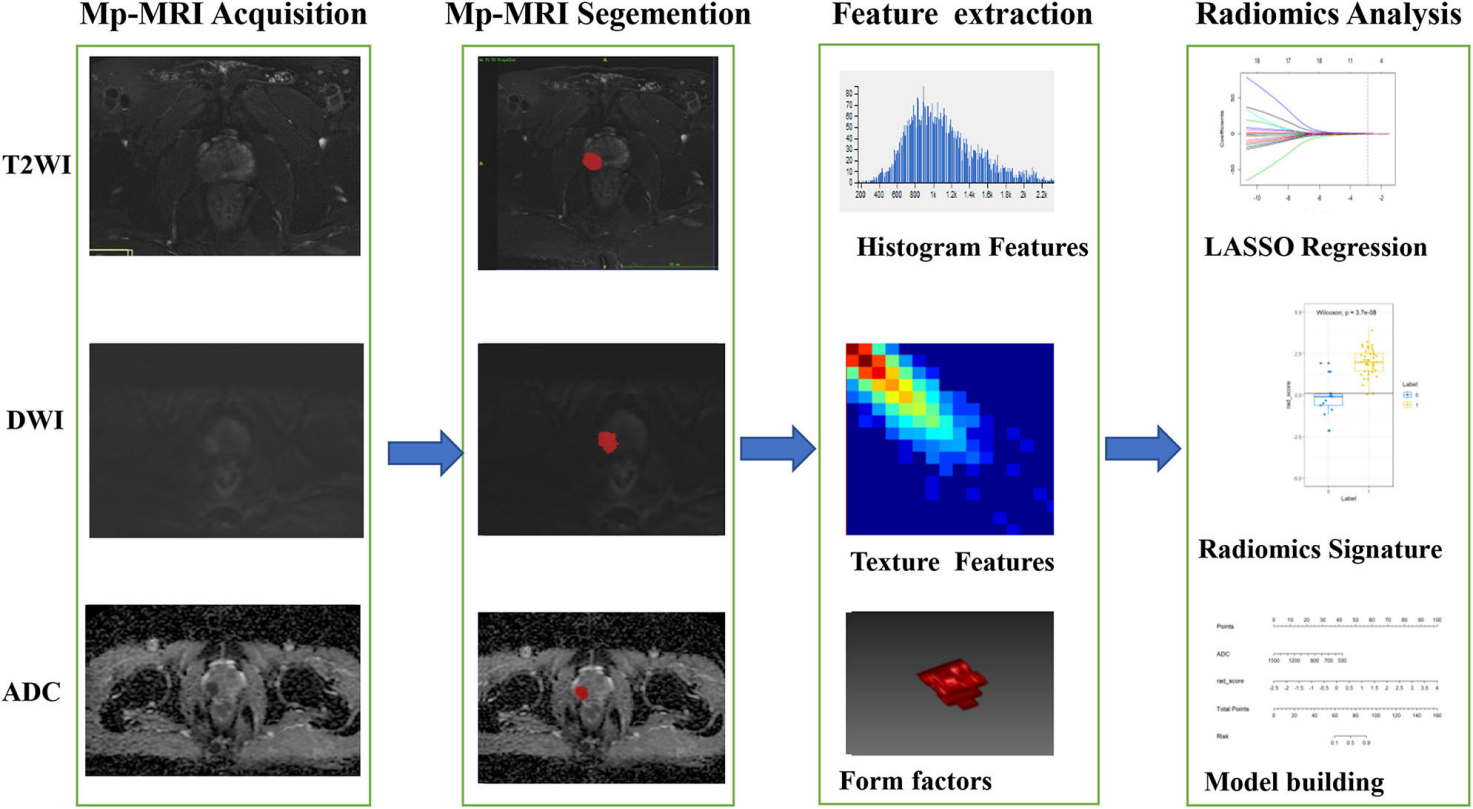
The framework for the radiomic workflow.

As the imbalance between csPCa and ciPCa patients may impact the classification capability, the synthetic minority over-sampling technique (SMOTE) was implemented in the training and validation group. Then, the two-feature selection method, minimum-redundancy maximum-relevance (mRMR), and least absolute shrinkage and selection operator (LASSO) were used to select the feature. At first, mRMR was performed to eliminate the redundant and irrelevant features; 20 features were retained. Then, LASSO was conducted to choose the optimized subset of features to construct the final model. Tenfold cross-validations were used to determine the optimal values of λ. Finally, only 10 of the most predictive features were chosen and the corresponding coefficients were evaluated. Predictive models were constructed by multivariable logistic regression with the selected 10 features. A Radiomic signature (Rad-score) was then calculated for each patient via a linear combination of selected features weighted by their respective coefficients in the predictive models. The radiomic workflow is demonstrated in Figure 2. The radiomics procedure is described in detail in Supplementary Material 2.

### Statistical Analysis

Categorical variables demonstrate the frequency, whereas continuous variables demonstrate the mean and standard deviation (SD). The Fisher's exact test or Chi-squared test was adopted to assess the categorical variables, when appropriate. The Mann–Whitney *U* test was implemented to analyze the non-normally distributed continuous variables. R software (v. 3.5.1, Vienna, Austria) and SPSS 22.0 (IBM, Armonk, NY) were used to perform statistical analysis. The LASSO logistic regression was utilized with the “glmnet” package. The receiver operating characteristic (ROC) plots were constructed by the “pROC” package. Delong test was used to compare statistical difference in AUC of patient discrimination among groups. The nomogram construction and calibration plotting were used by the “rms” package. The decision curve analysis curve plots were performed using the “rmda” package. The diagnostic efficacy of the predictor was evaluated using the values of accuracy, sensitivity, and specificity. A *P* < 0.05 in two-tailed analyses was used to define statistical significance.

## Results

### Clinical Characteristics of Patients

Table 1 highlights the patient's clinical characteristics. It showed no significant statistical difference in age (*p* = 0.054–0.700) and lesion location (*p* = 0.218–0.376), while the remaining parameters had statistical difference (*P* < 0.05). Univariate logistic analysis demonstrated the probability of csPCa having significant associations with the ADC value and PSA level, while other clinical factors were excluded (Table 2).

**Table 1:** Characteristics of patients in the training and validation groups.

**Characteristics**	**Training group**		***P***	**Internal validation group**		***P***	**External validation group**		***P***
	**csPCa (*n* = 41)**	**ciPCa (*n* = 13)**		**csPCa (*n* = 17)**	**ciPCa (*n* = 5)**		**csPCa (*n* = 62)**	**ciPCa (*n* = 21)**	
Age (years)	73.830 ± 9.423	72.080 ± 7.794	0.700	78.180 ± 9.488	71.200 ± 3.421	0.054	73.230 ± 9.074	70.570 ± 9.042	0.250
PSA (ng/ml)	78.870 ± 180.596	14.498 ± 17.249	0.009	135.778 ± 262.629	13.555 ± 11.726	0.046	51.768 ± 132.283	13.217 ± 7.969	0.026
ADC value	707.710 ± 78.221	844.020 ± 183.432	0.001	702.405 ± 89.633	835.680 ± 44.353	0.003	803.974 ± 106.950	885.545 ± 134.103	0.006
MRI T-stage			0.049			0.074			0.001
T2	26	13		8	5		26	19	
T3	9	NA		7	NA		26	2	
T4	6	NA		2	NA		10	NA	
Position			0.376			0.218			0.234
Peripheral zone	20	10		9	4		36	12	
Transitional zone	9	3		3	1		20	9	
Peripheral and Transitional zone	12	NA		5	NA		6	NA	
Gleason score			0.001			0.001			0.000
6	NA	13		NA	5		NA	21	
7	22	NA		9	NA		25	NA	
8	13	NA		6	NA		18	NA	
9	5	NA		2	NA		16	NA	
10	1	NA		NA	NA		3	NA	

*ADC, apparent diffusion coefficient; csPCa, clinically significant prostate cancer; ciPCa, clinically insignificant prostate cancer; NA, not available; PSA, prostate-specific antigen*.

**Table 2:** Logistic regression analyses for discriminating between clinically significant and clinically insignificant prostate cancer.

**Variable**	**Univariate logistic analysis**		**Multivariate logistic analysis**	
	**OR (95% CI)**	***P***	**OR (95% CI)**	***P***
MR-T stage	6.081 (2.1, 10)	0.991	NA^*^	NA^*^
Age	1.043 (0.980, 1.110)	0.183	NA^*^	NA^*^
ADC	0.983 (0.973, 0.992)	<0.001	0.985 (0.975, 0.995)	0.029
PSA	1.048 (1.007, 1.091)	0.022	1.024 (0.986, 1.064)	0.340

*ADC, apparent diffusion coefficient; CI, confidence interval; NA, not available; OR, odds ratio; PSA, prostate-specific antigen*.

* *These variables were eliminated in the multivariate logistic regression model. Therefore, the OR and P values were not available*.

The ADC value and PSA level were entered into multivariate logistic analysis. However, PSA was excluded due to a lack of significant differences (*p* = 0.340). The ADC value was lower in csPCa than in ciPCa and was the only remaining independent clinical risk factor (*p* = 0.022).

### Inter-observer and Intra-observer Agreement

The intra-observer ICC computed based on two extractions of reader 1 ranged from 0.827 to 0.934. The inter-observer agreement between two readers varied from 0.783 to 0.905. The results manifested high intra- and inter-observer feature extraction agreement.

### Radiomic Signature Development and Accuracy

**Figure 3:**
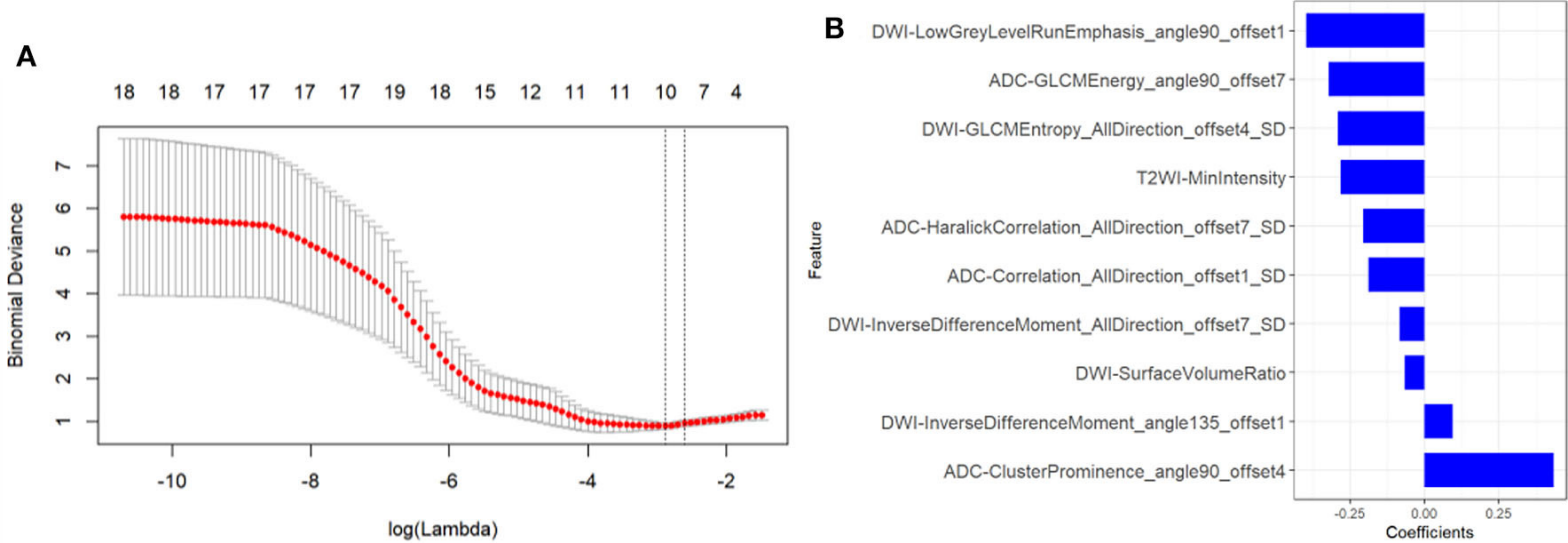
Texture feature selection. **(A)** Tuning parameter (λ) selection in the LASSO model used tenfold cross-validation via minimum criteria. The partial likelihood deviance was plotted versus log (λ). The dotted vertical lines were drawn at the optimal values using the minimum criteria and the 1-SE criteria. **(B)** The most predictive subset of feature was chosen and the corresponding coefficients were evaluated.

A total of 1,188 radiomic features were extracted from T2WI, DWI, and ADC imaging. During mRMR and LASSO processing, 10 radiomic features (5 from DW imaging, 4 from ADC imaging, and 1 feature from T2W imaging) were selected and were performed to build the radiomic signature (Figure 3). The values of the 10 selected features in each patient were input to the formula, and the rad-score was then acquired to reflect the probability of csPCa. The rad-score revealed a great predictive efficacy, with an AUC of 0.95 [95% confidence interval (CI), 0.87 to 1.0] in the training group and 0.86 (95% CI, 0.70 to 1.0) in the internal validation group. Furthermore, the AUC in external validation group achieved 0.81 (95% CI, 0.68 to 0.94).

### Development and Performance of the Radiomic Nomogram

The rad-score and ADC value were identified as independent predictors for discriminating between csPCa and ciPCa and then a radiomic nomogram was developed. Each independent predictor was allocated a weighted number of points. The overall number of points for each patient was computed using the nomogram and was associated with the likelihood of csPCa. The sensitivity, specificity, and accuracy of the radiomic signature and radiomic nomogram are demonstrated on Table 3.

**Table 3:** Predictive performance of the radiomic signature and radiomic nomogram.

**Model**	**Radiomic signature**		**Accuracy (95% CI)**	**ADC value**		**Accuracy (95% CI)**	**Radiomic nomogram**		**Accuracy (95% CI)**
	**Sensitivity**	**Specificity**		**Sensitivity**	**Specificity**		**Sensitivity**	**Specificity**	
Training group	0.846	0.976	0.944 (0.846–0.988)	0.935	0.478	0.741 (0.603–0.850)	0.952	0.916	0.944 (0.846–0.988)
Internal validation group	1.000	0.706	0.773 (0.546–0.921)	0.941	0.800	0.909 (0.708–0.989)	1.000	0.625	0.864 (0.651–0.971)
External validation group	0.800	0.727	0.786 (0.656–0.884)	0.756	0.636	0.732 (0.597–0.842)	0.771	1.000	0.798 (0.696–0.870)

*ADC, apparent diffusion coefficient; CI, confidence interval*.

**Figure 4:**
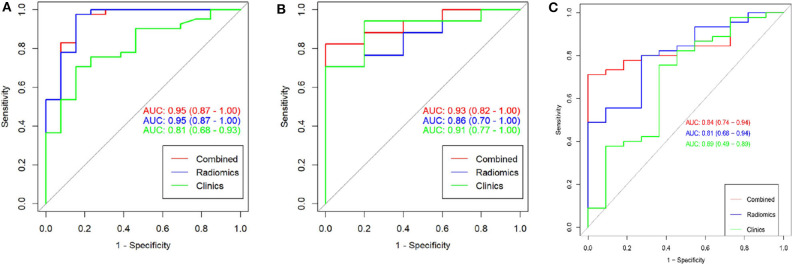
The receiver operating curves (ROC) of a combination nomogram, radiomic signatures, and clinical risk factor for discriminating clinically significant and clinically insignificant prostate cancer were presented in the training group **(A)**, internal validation group **(B)**, and external validation group **(C)**. The combination nomogram obtained the highest area under the curve (AUC).

**Figure 5:**
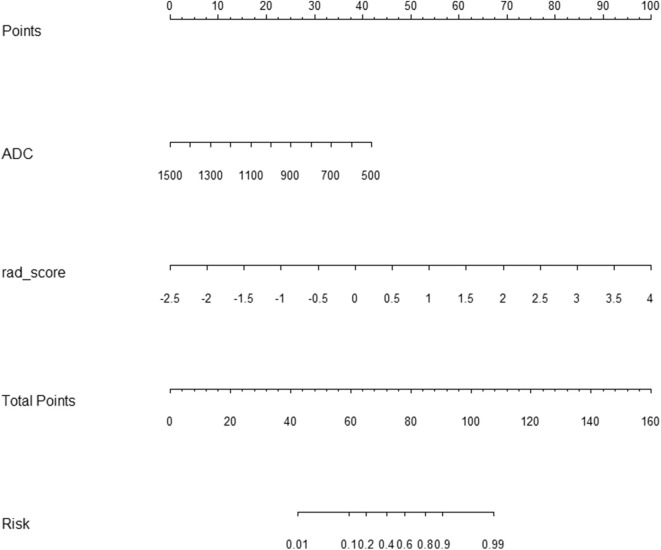
Radiomic nomogram to discriminate clinically significant and clinically insignificant prostate cancer. The radiomic nomogram was built on the training group, with the rad-score and ADC value. For example, a 74-year-old prostate cancer patient with an ADC value of 800 × 10^−6^ s/mm^2^, its radiomic signature score was 2, the total number of points of this tumor was 100 (30 + 70), and the risk rate of clinically significant prostate cancer was determined to be 90%. ADC, apparent diffusion coefficient.

To compare the discrimination performance, the ROC curves were plotted for radiomic nomogram, rad-score, and ADC value in the training group. The radiomic nomogram demonstrated a favorable classification capability with the AUC of 0.95 (training group), 0.93 (internal validation group), and 0.84 (external validation group) (Figures 4A–C). Therefore, the nomogram was superior to the rad-score and ADC value alone in discriminating csPCa from ciPCa, especially in the internal and external validation group. Details of the performance of radiomic nomogram are shown in Figure 5. Delong test was performed to verify the statistical difference in AUC of patient discrimination between nomogram, rad-score, and ADC score. This result was presented in Supplementary Table 2.

**Figure 6:**
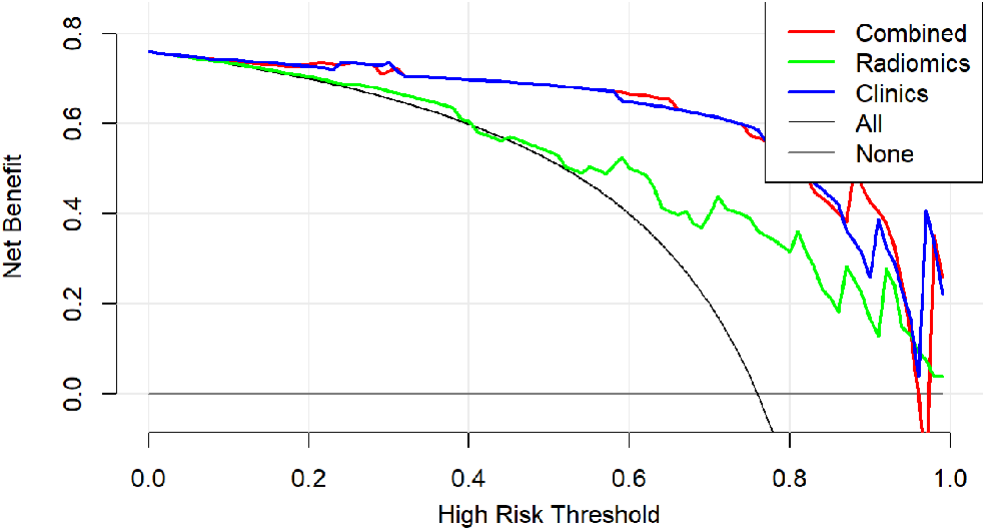
Decision curve analysis of clinical use assessment of the radiomic nomogram in the validation group. The *Y*-axis represented the net benefit. The method was the best for feature selection if it had the highest net benefit. The radiomic nomogram (red line) achieved the highest net benefit compared with the radiomic signature (green line), clinical characteristics (blue line), treat-all strategy (gray line), and the treat-none strategy (horizontal black line).

Finally, a decision curve analysis was performed to evaluate whether this nomogram would assist in differentiating between csPCa from ciPCa (Figure 6). When the threshold probability ranged from 0 to 1 according to the decision curve analysis, the nomogram obtained the greatest benefit compared with a “treat all” strategy, a “treat none” strategy.

## Discussion

This study developed and validated a radiomic nomogram for discriminating between csPCa and ciPCa in the present study. The nomogram was constructed by containing the rad-score from the radiomic method and ADC value. Rad-score was described as the probability of csPCa computed from the radiomic signature, which was built based on 10 selective radiomic features. Both the radiomic signature and nomogram demonstrated the same capability to discriminate between csPCa and ciPCa in the training group (AUC = 0.95 vs. 0.95). However, the nomogram exceeded the radiomic model in the internal (AUC = 0.93 vs. 0.86) and external (AUC = 0.84 vs. 0.81) validation group. Thus, the results shown herein indicate that the radiomic model may serve as a potential non-invasive method to differentiate between csPCa and ciPCa in clinical practice.

Recently, radiomics has been successfully applied in oncology and extended to PCa identification and evaluation (15, 23–25). Chen et al. compared a radiomic-based model with PI-RADS v2 scores in differentiating and grading PCa (15). This result suggested that radiomic models offered a high diagnostic accuracy and outperformed the corresponding PI-RADS v2 scores. Min et al. investigated an mp-MRI-based radiomic signature for identifying csPCa with an AUC of 0.823 in the validation group (18). The AUC of the radiomic signature for predicting csPCa was 0.86 (internal validation group) and 0.84 (external validation group) in our study, which differed from the result provided by Min et al. The difference may be illustrated by differences in research populations and patient selection criteria. In addition, our study incorporated the ADC value, PSA level, MR-T stage, and age. These parameters were included as they are of great importance in differentiating csPCa in clinical settings. The nomogram constructed from the aforementioned features may provide an individualized evaluation of csPCa. Our results suggested that the radiomic nomogram had a great efficacy for prediction csPCa in both training group and internal and external validation groups (AUC = 0.95, 0.93, and 0.84, respectively).

In our present study, the overall 1,188 radiomic features were extracted from T2WI, DWI, and ADC imaging. In total, 10 radiomic features were selected. Of these, nine radiomic features were derived from DWI and ADC imaging, including six texture features, two form factor features, and one histogram feature. The mostly radiomic features selected in this study were texture features about the statistical correlation between local nearby voxels with similar (or dissimilar) contrast values (26). This indicated that radiomic signature could support a prebiopsy potential in differentiating between csPCa and ciPCa.

ADC value was the only risk factor found in all clinical risk factors. The performance of both the radiomic signature and ADC value were high and comparable in the validation group in our study. This is consistent with a recent report with radiomic machine learning, which showed similar results (27). It may be the result of the principal nature of DWI and ADC that could dramatically reflect PCa pathological status in the peripheral zone. Indeed, most of PCa lesions lay in the peripheral zone in our study. DWI and more specifically ADC have been regarded as the most powerful sequence of prostate MR, especially in the peripheral zone (28). ADC values have been suggested to be reproducible quantitative markers to evaluate PCa aggressiveness (29, 30).

It is worth noting that the PSA level widely used in the PCa detection was not a significant factor regarding the differentiation of csPCa, which makes the elimination of this variable for model development. It is likely explained that the PSA level is specific to prostate tissue but not to PCa lesion. Another explanation may be related with the nuances in the data group or confounding by other risk factors. MR-T stage demonstrating the highest odds ratio value was also excluded to build the predictive model in our study. This finding probably associates with the extension degree of csPCa lesions. When csPCa lesions did not present with invasion of extra prostate capsular tissues, such as the neurovascular bundle, seminal vesicles, and distal sphincter, the MR-T was ascribed to the T2 stage. Obviously, the MR-T stage of all ciPCa patients was ascribed to the T2 stage.

The ratio of the csPCa and ciPCa patients was different (120 vs. 39) in the present study. This inter-group imbalance may give rise to bias for the build radiomic signatures in the training group, which would impact the prediction capability of the radiomic signature in the validation group. To reduce the effect of the imbalance, the SMOTE algorithm was applied to construct the radiomic model. However, the performance of the training and validation group was still in agreement with our original data and sample size. The quality assurance of the MRI scanner should also be illustrated. The present material spanned up to 3 years, so the imaging quality of the MRI scanner was essential to maintaining rigor to the long duration of this study. Therefore, the quality assurance maintenance records of the MRI scanner were reviewed and approved.

Several limitations to the current study should be noted. First, the current study has a small sample size and is a retrospective study from two centers. Therefore, large sample sizes from multiple centers are necessary to validate our primary findings. Second, systematic biopsy was applied for the pathological standard instead of the whole-mount pathological specimen. The experienced radiologists exerted all efforts to match the MRI lesion and the pathological site. It is obviously unreasonable that all of our subjects would have the whole-mount pathological specimen, especially for ciPCa patients. Moreover, patients with a lesion diameter of less than 5 mm on mp-MRI images were eliminated because we could not outline the PCa region during MRI segmentation. This may cause patient selection bias. Although our methodical strategies have a few limitations, we hold the view that they supply ample verification for the principal findings of our primary study.

In conclusion, this study presents a radiomic nomogram that incorporates both the radiomic signature and clinical risk factors for discriminating csPCa from ciPCa. The nomogram, incorporating radiomic signature and ADC value, provided an individualized, potential approach for discriminating csPCa from ciPCa. Further studies with large sample sizes from multiple centers are necessary to validate our primary results. With further investigation, it is possible that this radiomic nomogram may aid clinicians in determining prebiopsy and pre-treatment risk stratification for PCa.

## Data Availability Statement

The datasets generated for this study are available on request to the corresponding author.

## Ethics Statement

The studies involving human participants were reviewed and approved by The Ethics Committee of the Guangxing Hospital Affiliated to Zhejiang Chinese Medical University. Written informed consent for participation was not required for this study in accordance with the national legislation and the institutional requirements.

## Author Contributions

FC and MX conceived and launched this study. YZ, WC, and JS recruited patients and acquired clinical information. XY conducted the quality assurance of image quality. CG and PP analyzed the data and performed statistical experiments. YZ and PP provided result interpretation. YZ wrote the first draft of this manuscript. FC, MX, and PP revised and edited the final version. WC, JS, XY, CG, and FC reviewed the manuscript. All authors contributed to the article and approved the submitted version.

## Conflict of Interest

PP was employed by GE Healthcare Life Sciences. The remaining authors declare that the research was conducted in the absence of any commercial or financial relationships that could be construed as a potential conflict of interest.

## References

[1] Bray F Ferlay J Soerjomataram I Siegel RL Torre LA Jemal A. Global cancer statistics 2018: GLOBOCAN estimates of incidence and mortality worldwide for 36 cancers in 185 countries. Cancer J Clin. (2018) 6:394–424. 10.3322/caac.21492 30207593

[2] Mottet N Bellmunt J Bolla M Briers E Cumberbatch MG De Santis M. EAU-ESTRO-SIOG guidelines on prostate cancer. Part 1: screening, diagnosis, and local treatment with curative intent. Eur Urol. (2017) 71:618–29. 10.1016/j.eururo.2016.08.003 27568654

[3] Rosario DJ Lane JA Metcalfe C Donovan JL Doble A Goodwin L. Short term outcomes of prostate biopsy in men tested for cancer by prostate specific antigen: prospective evaluation within ProtecT study. BMJ. (2012) 344:d7894. 10.1136/bmj.d7894 22232535 PMC3253765

[4] Ahmed HU El-Shater Bosaily A Brown LC Gabe R Kaplan R Parmar MK Emberton, diagnostic accuracy of multi-parametric MRI and TRUS biopsy in prostate cancer (PROMIS): a paired validating confirmatory study. Lancet. (2017) 389:815–22. 10.1016/S0140-6736(16)32401-1 28110982

[5] Mehralivand S Shih JH Rais-Bahrami S Oto A Bednarova S Nix JW. A magnetic resonance imaging-based prediction model for prostate biopsy risk stratification. JAMA Oncol. (2018) 4:678–85. 10.1001/jamaoncol.2017.5667 29470570 PMC5885194

[6] Hausmann D Aksoz N von Hardenberg J Martini T Westhoff N Buettner S. Prostate cancer detection among readers with different degree of experience using ultra-high b-value diffusion-weighted imaging: Is a noncontrast protocol sufficient to detect significant cancer? Eur Radiol. (2018) 28:869–76. 10.1007/s00330-017-5004-8 28799090

[7] Weinreb JC Barentsz JO Choyke PL Cornud F Haider MA Macura KJ. PI-RADS prostate imaging - reporting and data system: 2015, version 2. Eur Urol. (2016) 69:16–40. 10.1016/j.eururo.2015.08.052 26427566 PMC6467207

[8] Thompson JE van Leeuwen PJ Moses D Shnier R Brenner P Delprado W. The diagnostic performance of multiparametric magnetic resonance imaging to detect significant prostate cancer. J Urol. (2016) 195:1428–35. 10.1016/j.juro.2015.10.140 26529298

[9] Greer MD Brown AM Shih JH Summers RM Marko J Law YM. Accuracy and agreement of PI-RADS v2 for prostate cancer mpMRI: a multireader study. J Magn Reson Imaging. (2017) 45:579–85. 10.1002/jmri.25372 27391860 PMC7900895

[10] Chen F Cen S Palmer S Application of prostate imaging reporting and data system version 2 (PI-RADS v2): inter-observer agreement and positive predictive value for localization of intermediate- and high-grade prostate cancers on multiparametric magnetic resonance imaging. Acad Radiol. (2017) 24:1101–6. 10.1016/j.acra.2017.03.019 28546032

[11] Gillies RJ Kinahan PE Hricak H. Radiomics: images are more than pictures, they are data. Radiology. (2015) 278:563–77. 10.1148/radiol.2015151169 26579733 PMC4734157

[12] Huang YQ Liang CH He L Tian J Liang CS Chen X. Development and validation of a radiomics nomogram for preoperative prediction of lymph node metastasis in colorectal cancer. J Clin Oncol. (2016) 34:2157–64. 10.1200/JCO.2015.65.9128 27138577

[13] Li H Zhu Y Burnside ES Drukker K Hoadley KA Fan C. MR imaging radiomics signatures for predicting the risk of breast cancer recurrence as given by research versions of MammaPrint, oncotype DX, and PAM50 gene assays. Radiology. (2016) 281:382–91. 10.1148/radiol.2016152110 27144536 PMC5069147

[14] Dong D Tang L Li ZY Fang MJ Gao JB Shan XH. Development and validation of an individualized nomogram to identify occult peritoneal metastasis in patients with advanced gastric cancer. Ann Oncol. (2019) 30:431–8. 10.1093/annonc/mdz001 30689702 PMC6442651

[15] Chen T Li M Gu Y Zhang Y Yang S Wei C. Prostate cancer differentiation and aggressiveness: assessment with a radiomic-based model vs. PI-RADS v2. J Magn Reson Imaging. (2019) 49:875–84. 10.1002/jmri.26243 30230108 PMC6620601

[16] Sidhu HS Benigno S Ganeshan B Dikaios N Johnston EW Allen C. Textural analysis of multiparametric MRI detects transition zone prostate cancer. Eur Radiol. (2017) 27:2348–58. 10.1007/s00330-016-4579-9 27620864 PMC5408048

[17] Wibmer A Hricak H Gondo T Matsumoto K Veeraraghavan H Fehr D. Haralick texture analysis of prostate MRI: utility for differentiating non-cancerous prostate from prostate cancer and differentiating prostate cancers with different Gleason scores. Eur Radiol. (2015) 25:2840–50. 10.1007/s00330-015-3701-8 25991476 PMC5026307

[18] Min X Li M Dong D Feng Z Zhang P Ke Z. Multi-parametric MRI-based radiomics signature for discriminating between clinically significant and insignificant prostate cancer: Cross-validation of a machine learning method. Eur J Radiol. (2019) 115:16–21. 10.1016/j.ejrad.2019.03.010 31084754

[19] Xu L Yang P Liang W Liu W Wang W Luo C. A radiomics approach based on support vector machine using MR images for preoperative lymph node status evaluation in intrahepatic cholangiocarcinoma. Theranostics. (2019) 9:5374–85. 10.7150/thno.34149 31410221 PMC6691572

[20] Carroll PH Mohler JL. NCCN Guidelines updates: prostate cancer and prostate cancer early detection. J Natl Compr Canc Netw. (2018) 16:620–3. 10.6004/jnccn.2018.0036 29784740

[21] Baki MM Menys A Atkinson D Bassett P Morley S. Feasibility of vocal fold abduction and adduction assessment using cine-MRI. Eur Radiol. (2017) 27:598–606. 10.1007/s00330-016-4341-3 27085701 PMC5209431

[22] Chen J Chen Y Zheng D Pang P Lu J Zheng X. Pretreatment MR-based radiomics signature as potential imaging biomarker for assessing the expression of topoisomerase ii α (topo-iiα) in rectal cancer. J Magn Reson Imaging. (2020) 51:1881–9. 10.1002/jmri.26972 31675149

[23] Chaddad A Niazi T Probst S Bladou F Anidjar M Bahoric B. Predicting gleason score of prostate cancer patients using radiomic analysis. Front Oncol. (2018) 8:630. 10.3389/fonc.2018.00630 30619764 PMC6305278

[24] Shiradkar R Ghose S Jambor I Taimen P Ettala O Purysko AS. Radiomic features from pretreatment biparametric MRI predict prostate cancer biochemical recurrence: preliminary findings. J Magn Reson Imaging. (2018) 48:1626–36. 10.1002/jmri.26178 29734484 PMC6222024

[25] Jiang Y Chen C Xie J Wang W Zha X Lv W. Radiomics signature of computed tomography imaging for prediction of survival and chemotherapeutic benefits in gastric cancer. EBioMedicine. (2018) 36:171–82. 10.1016/j.ebiom.2018.09.007 30224313 PMC6197796

[26] Chen W Wang S Dong D Gao X Zhou K Li J. Evaluation of lymph node metastasis in advanced gastric cancer using magnetic resonance imaging-based radiomics. Front Oncol. (2019) 9:1265. 10.3389/fonc.2019.01265 31824847 PMC6883384

[27] Bonekamp D Kohl S Wiesenfarth M Schelb P Radtke JP Götz M. Radiomic machine learning for characterization of prostate lesions with MRI: comparison to ADC values. Radiology. (2018) 289:128–37. 10.1148/radiol.2018173064 30063191

[28] Chatterjee A Watson G Myint E Sved P McEntee M Bourne R. Changes in epithelium, stroma, and lumen space correlate more strongly with gleason pattern and are stronger predictors of prostate ADC changes than cellularity metrics. Radiology. (2015) 277:751–62. 10.1148/radiol.2015142414 26110669

[29] Hoang Dinh A Melodelima C Souchon R Lehaire J Bratan F Mège-Lechevallier F. Quantitative analysis of prostate multiparametric MR images for detection of aggressive prostate cancer in the peripheral zone: a multiple imager study. Radiology. (2016) 280:117–27. 10.1148/radiol.2016151406 26859255

[30] Peng Y Jiang Y Antic T Giger ML Eggener SE Oto A. Validation of quantitative analysis of multiparametric prostate MR images for prostate cancer detection and aggressiveness assessment: a Cross-imager study. Radiology. (2014) 271:461–71. 10.1148/radiol.14131320 24533870

