# Supplementary material for: Development of a Novel, Multi-Parametric, MRI-Based Radiomic Nomogram for Differentiating Between Clinically Significant and Insignificant Prostate Cancer
Source: Front Oncol. 2020 Jun 30;10:888. doi: 10.3389/fonc.2020.00888 (PMC7339043; doi:10.3389/fonc.2020.00888)
Supplement: Supplementary file 1 [file Data_Sheet_1.docx]

Supplementary Material

# 1 Supplementary Figures and Tables

## 1.1 Supplementary Figures

**Supplementary Table 1**. These details of the imaging sequence parameters for two center

| The Guangxing Hospital Affiliated to Zhejiang Chinese Medical University | | | | |
| --- | --- | --- | --- | --- |
| Parameters | T1WI | T2WI | DWI | DCE |
| Sequence | FSE | FSE | EPI | LAVA |
| TR/TE (ms) | 843/12.2 | 13300/100 | 3232/57.3 | 2.6/1.1 |
| Slice thickness (mm) | 5 | 3 | 3 | 3 |
| Gap (mm) | 1 | 1 | 1.5 | 0 |
| FOV (cm) | 38×38 | 20×20 | 40×32 | 38×38 |
| Matrix | 320×192 | 288×288 | 128×128 | 200×160 |
| The First Affiliated Hospital of Zhejiang Chinese Medical University | | | | |
| Parameters | T1WI | T2WI | DWI | DCE |
| Sequence | FSE | FSE | EPI | LAVA |
| TR/TE (ms) | 795/11.2 | 12900/100 | 3541/67.3 | 3.7/1.4 |
| Slice thickness (mm) | 5 | 3.5 | 3.5 | 3.5 |
| Gap (mm) | 1 | 1 | 1.5 | 0 |
| FOV (cm) | 40×4 | 18×20 | 38×32 | 40×40 |
| Matrix | 320×192 | 288×288 | 128×128 | 200×160 |

**Supplementary Table 2.**  The statistical difference in AUC of patient discrimination between nomogram, rad-score and ADC score by Delong test.

| Comparison (p value) | nomogram vs. rad-score | nomogram vs. ADC score | rad-score vs. ADC score |
| --- | --- | --- | --- |
| Training group | 0.778 | 0.023 | 0.033 |
| Internal validation group | 0.168 | 0.738 | 0.673 |
| External validation group | 0.505 | 0.081 | 0.046 |


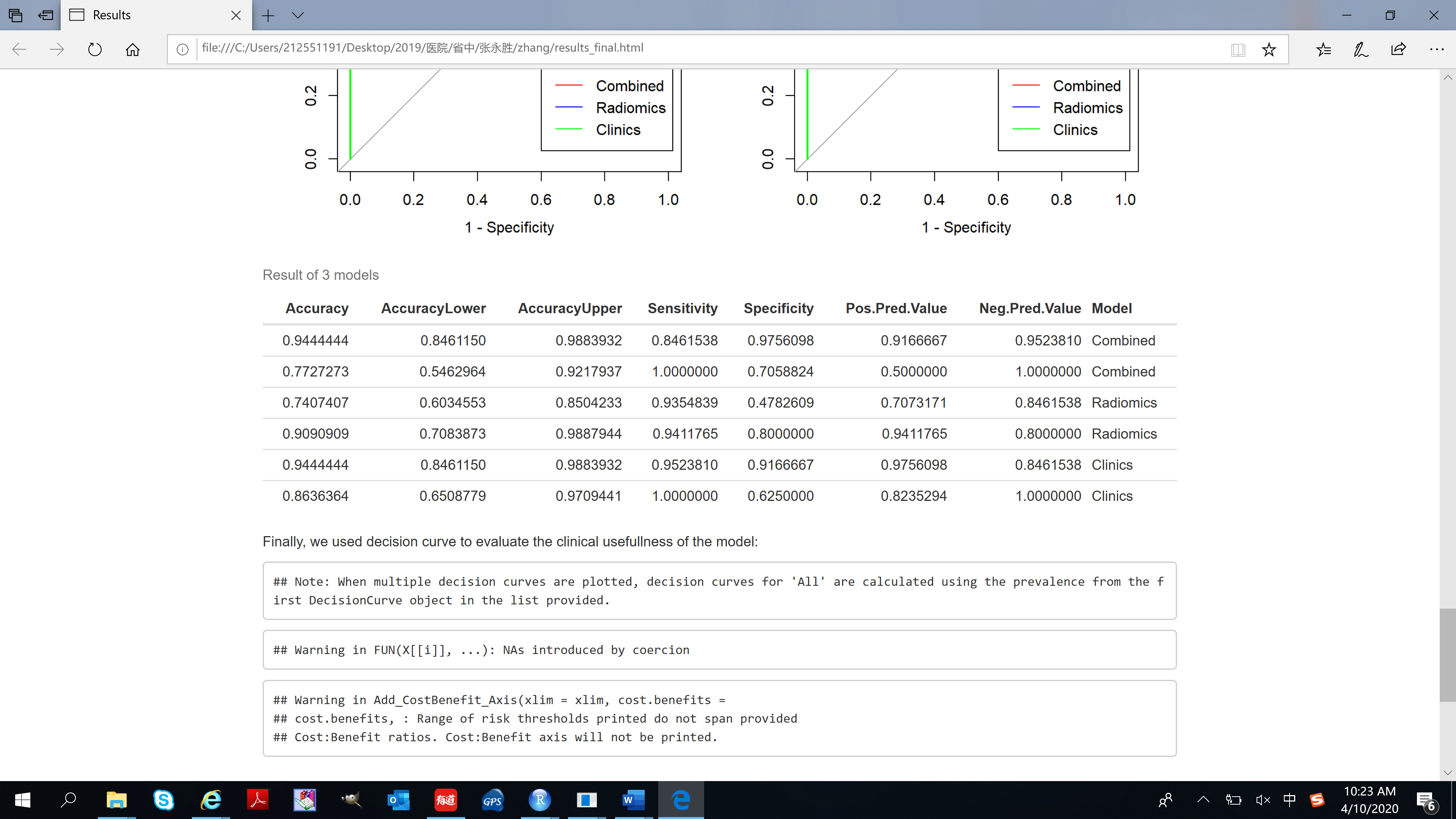


Supplementary figure 1. The detail predictive performance of the radiomic signature and radiomic nomogram.The first and second line was the Radiomics model, the third and fourth line was Clinics model, the last two line was Combined model.


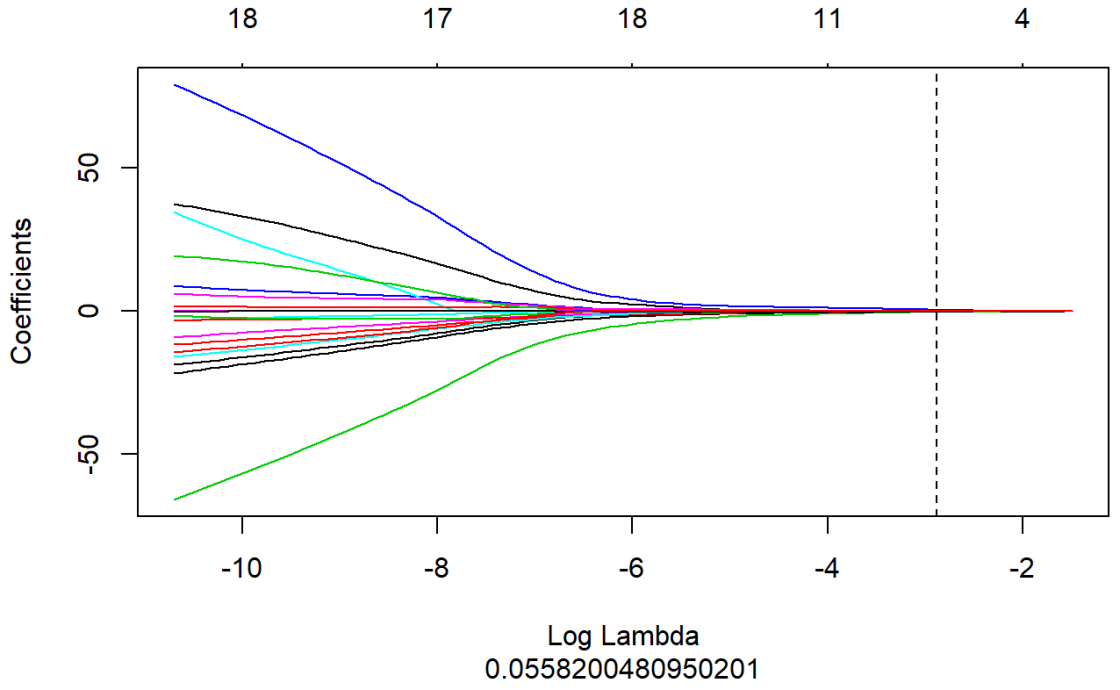


Supplementary figure 2. LASSO coefficient profiles of texture features. A vertical line as drawn at the value selected using 10-fold cross-validation in log (λ) sequence and displayed ten coefficients with non-zero values.

**2. Supplementary Methods**

**2.1. The radiomics procedure**

- 1. **Image preprocessing**

All MRI images data were exported from the PACS system in the original DICOM format, and then converted to NIFTI format by MRIcron (https://www.nitrc.org/projects/mricron). Firstly, all MRI images were resampled to voxels of 1×1×1mm, which was helpful for the calculation of texture features, then gauss filtering was used to reduce noise and skull removal and magnetic field migration correction were performed, which helped to reduce external interference factors. Finally, the gray level of all images were normalized to 0-255 gray level, which was helpful to the comparative analysis of textures.

2.2 Data dimension reduction methodology

Firstly, extracted texture features were standardized(z-score), which could remove the unit limits of the data of each feature so that the indexes of different units or orders could be compared and weighted. Then the feature dimensionality reduction was performed as follows. The analysis of variance (ANOVA) and Mann-Whitney U test (MW) dimensionality reduction were performed, and then the correlation test was calculated to reduce data redundancy. The software calculated the paired correlation between each two of the features. If the Spearman correlation coefficient was greater than 0.9, it was approach to the linear relationship, in other words, the two features could express each other, which had no effect to the result, but the coefficient was different when fitting. In this study, the software automatically removed the feature of the latter when the two features were relatively relevant. All the above steps were carried out by AK software. Figure S1 showed the flowchart of radiomics features dimension reduction. Table S1 showed the classification and calculation formula of texture features after dimension reduction.

z-score normalization to make the image intensities have the properties of a standard normal distribution with $\mu\text{=}1$ and $\sigma\text{=}0$, where $\mu$ was the mean value of the images, and was the standard deviation. The normalized values (also called z scores) of the image intensities (*x*) were $\sigma$calculated as follows:

$$z=\frac{x-\mu}{\sigma}$$

**2.3 Detailed descriptions of the statistical methodology and feature**

**2.3.1 The least absolute shrinkage and selection operator (LASSO) algorithm**

LASSO is a powerful algorithm for regression analysis with high dimensional predictors. In our study, the LASSO algorithm was combined with the logistic regression model for model development. We used the LASSO logistic regression model to select the most important predictive features and construct a radiomics signature in the training set. This algorithm minimizes a log partial likelihood subject to the sum of the absolute values of the parameters bounded by a constant:

subject to $\sum\left|\beta_{j}\right|\leq t$

where $\beta^{\wedge}$is the obtained parameters, is the log partial likelihood of the logistic regression model, and $t>0$is a constant.

The LASSO algorithm shrinks some coefficients and reduces others to exactly 0 via the absolute constraint. Thus, LASSO is an outstanding method for feature selection by retaining the good features of both subset selection and ridge regression. In this study, the constant *t* was set as 0.015, and LASSO selected nonzero coefficients`1$\beta^{\wedge}$, and a formula was generated using a linear combination of selected features that were weighted by their respective LASSO coefficients. The “glmnet” package in R statistical software version 3.4.1 was used for LASSO logistic regression model analysis.

**2.3.2 The minimum-redundancy maximum -relevance (mRMR) algorithm**

**
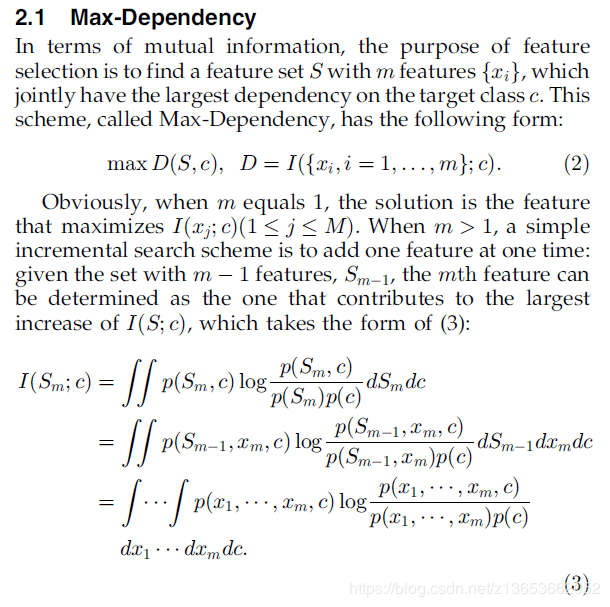
**

**2.3.4** Radiomics signature construction

Radscore was calculated by summing the selected features weighted by their coefficients. The final formula of radscore is:## [1] "Radscore = -0.066*DWI-SurfaceVolumeRatio+-0.083*DWI-InverseDifferenceMoment_AllDirection_offset7_SD+-0.321*ADC-GLCMEnergy_angle90_offset7+-0.188*ADC-Correlation_AllDirection_offset1_SD+-0.29*DWI-GLCMEntropy_AllDirection_offset4_SD+-0.205*ADC-HaralickCorrelation_AllDirection_offset7_SD+-0.397*DWI-LowGreyLevelRunEmphasis_angle90_offset1+0.434*ADC-ClusterProminence_angle90_offset4+-0.281*T2WI-MinIntensity+0.094*DWI-InverseDifferenceMoment_angle135_offset1 + 1.473".

**2.3.5 Details of the GLCM and RLM**

**Co-occurrence matrices**

The Grey level co-occurrence matrix (GLCM) $P(\left.i,j\right|\theta,d)$represents the joint probability of certain sets of pixels having certain grey-level values. It calculates how many times a pixel with grey-level $i$occurs jointly with another pixel having a grey value $j$. By varying the displacement vector $d$between each pair of pixels.

The advantage of the co-occurrence matrix calculations is that the co-occurring pairs of pixels can be spatially related in various orientations with reference to distance and angular spatial relationships, as on considering the relationship between two pixels at a time. As a result, the combination of grey levels and their positions are exhibited apparently. Therefore, it is defined as “A two-dimensional histogram of gray levels for pair of pixels, which are separated by a fixed spatial relationship”. However, the matrix is sensitive to rotation. With the change of different offsets define pixel relationships by varying directions.

The rotation angle of an offset: $0^{\text{°}},45^{\text{°}},90^{\text{°}},135^{\text{°}}$and displacement vectors (distance to the neighbor pixel: 1, 2, 3 ...), different co-occurrence distributions from the same image of reference. GLCM of an image is computed using displacement vector d defined by its radius, (distance or count to the next adjacent neighbor preferably is equal to one) and rotational angles.


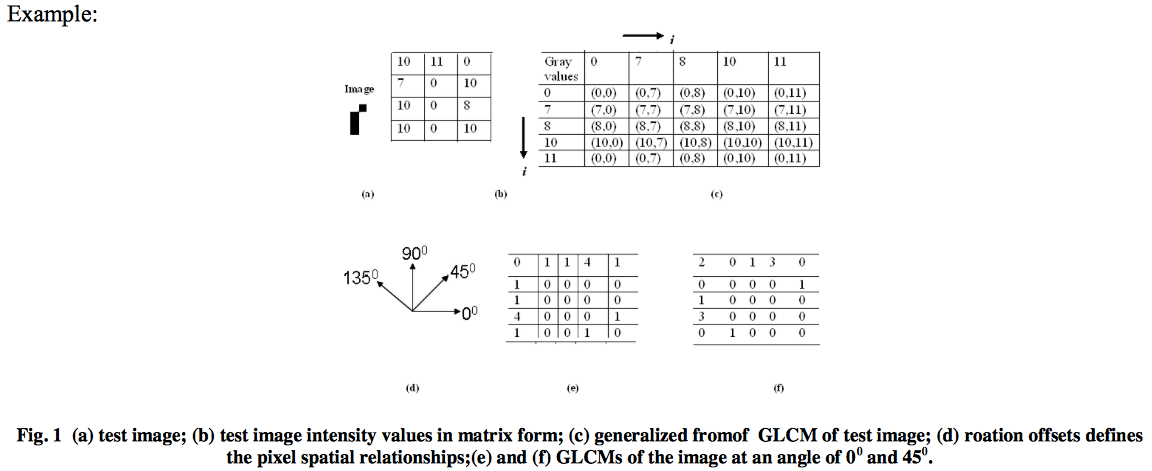


**Run-length matrices**

### The grey level run-length matrix (RLM) $P_{r}(\left.i,j\right|\theta)$is defined as the numbers of runs with pixels of gray level *i* and run length *j* for a given direction$\theta$. RLMs is generated for each sample image segment having directions (0°,45°,90° &135°), then the following ten statistical features were derived: short run emphasis, long run emphasis, grey level non-uniformity, run length non-uniformity, Low Grey Level Run Emphasis, High Grey Level Run Emphasis, Short Run Low Grey Level Emphasis, Short Run High Grey Level Emphasis, Long Run Low Grey Level Emphasis and Long Run High Grey Level Emphasis.
